# Greenhouse Gas Emissions from Large-Scale Dairy Farms: Interannual Dynamics, Farm-Level Heterogeneity, and Key Determinants

**DOI:** 10.3390/ani16203248

**Published:** 2026-10-15

**Authors:** Yueyu Shen, Xiaodong Su, Yadong Ma, Yunlong Liu, Xin Zhang, Zeeshan Muhammad, Sana Asif, Di Pei, Yan Tu

**Affiliations:** 1 Institute of Feed Research, Chinese Academy of Agricultural Sciences/Key Laboratory of Feed Biotechnology of the Ministry of Agriculture and Rural Affairs, Beijing 100081, China; lcxmmyl@126.com (Y.S.); 17899314068@163.com (Y.M.); liuyunlong@caas.cn (Y.L.); nixgnahz@126.com (X.Z.); 2024y90100025@caas.cn (Z.M.); sanaasif386@gmail.com (S.A.); 2 College of Agriculture and Animal Husbandry, Qinghai University, Xining 810016, China; 3 Beijing Sunlon Livestock Development Company Limited, Beijing 100176, China; lotus.pei@icloud.com; 4 Beijing Innovation Consortium of Livestock Research System (BAIC05), Beijing 100081, China

**Keywords:** greenhouse gas footprint, carbon emission intensity, life cycle assessment, interannual variability, manure management

## Abstract

The transition to low-emission (LE) dairy production is essential for mitigating agricultural greenhouse gas (GHG) emissions. However, the interannual variability and principal drivers of farm-level GHG footprints across intensive dairy farms remain insufficiently characterized, constraining targeted mitigation strategies. This study aimed to (i) quantify interannual variation in on-farm GHG emission intensity, (ii) characterize management contrasts between high- and low-emission farms, (iii) clarify the underlying mechanisms, and (iv) identify optimization pathways. A supplementary sensitivity analysis encompassing upstream emissions associated with feed production was performed using an expanded system boundary to assess the effects of system boundary selection on the estimated GHG emissions. Using 3-year (2021–2023) field data from 22 intensive dairy farms, a standardized farm-level GHG inventory was used to quantify operational GHG emission intensity per unit of fat- and protein-corrected milk (FPCM). The production-weighted mean on-farm operational GHG intensity was 0.86 kg CO_2_-eq kg^−1^ FPCM, with enteric methane constituting the dominant emission source. Incorporating upstream emissions associated with feed production contributed an additional 0.427 kg CO_2_-eq kg^−1^ FPCM, yielding a total emission intensity of 1.287 kg CO_2_-eq kg^−1^ FPCM under the expanded system boundary, representing a 49.7% increase relative to the operational inventory. Feed upstream emissions accounted for 33.2% of the total emissions estimated within the expanded system boundary. Both herd size and energy consumption exhibited increasing trends throughout the study period, whereas the proportion of lactating cows relating to the total herd declined from 46.01% in 2021 to 44.32% in 2023, suggesting that the expansion of the overall herd exceeded the corresponding growth in the lactating cow population. Under the operational system boundary, high-emission (HE) farms exhibited relatively greater contributions to their GHG emission profiles from enteric methane production, electricity consumption, and manure management activities. Conversely, LE farms attained improved performance through higher proportions of lactating cows, greater feed efficiency, and manure anaerobic digestion. Correlation analysis linked herd size, energy consumption, and dry matter intake positively to on-farm GHG emissions. Dietary starch was associated with manure N_2_O emissions, while feed efficiency was associated with higher energy-related GHG emissions. Overall, herd composition, feeding management practices, energy utilization, and manure management strategies were identified as principal determinants of on-farm GHG emission performance. Furthermore, the sensitivity analysis employing an expanded system boundary demonstrated that incorporating upstream emissions associated with feed production substantially elevates the overall GHG emission intensity expressed per kg FPCM. This study helps address the lack of multi-year farm-level evidence for China’s dairy sector and proposes integrated strategies—herd structure adjustment, precision feeding, clean energy exploitation, and manure management—alongside sustained long-term monitoring, given the pronounced interannual variability. These findings highlight the roles of farm-level GHG inventories in identifying targeted on-farm emission mitigation opportunities and expanded system boundary assessments in quantifying the overall GHG burden associated with the entire dairy production supply chain.

## Simple Summary

Dairy farming constitutes a critical contributor to agricultural greenhouse gas (GHG) emissions, and mitigating the carbon footprint of milk production is essential to achieve global climate mitigation targets. Nevertheless, the key factors driving inter-farm discrepancies in GHG emissions and the temporal variability in such emissions across different years remain poorly understood. In this work, a three-year field investigation was conducted on 22 large-scale dairy farms located in North China to quantify on-farm GHG emission characteristics. The results showed that the average emission intensity reached 0.86 kg CO_2_-equivalent per kilogram of fat- and protein-corrected milk (FPCM), with enteric methane from ruminant digestion representing the dominant source of total GHG emissions. Significant interannual variations in GHG outputs were observed across the surveyed farms, demonstrating that long-term continuous monitoring is indispensable, rather than relying solely on one-year static evaluation data. Notably, low-emission dairy farms exhibited superior production performance with a higher milk yield and a lower carbon output. The advantages of these efficient farms were mainly reflected in a higher proportion of lactating cows in the herd, improved feed utilization efficiency, and the adoption of anaerobic digestion technology for manure disposal. Collectively, this study confirms that the optimization of herd structure, refinement of precision feeding management, and upgrading of manure treatment systems can effectively reduce on-farm GHG emissions without compromising milk productivity. These outcomes provide feasible technical and managerial references for the low-carbon and sustainable development of the dairy industry.

## 1. Introduction

Milk and dairy products are common sources of protein consumed by over 6 billion people worldwide, underscoring the broad global reliance on milk and dairy products [1]. However, the greenhouse gases (GHGs) (e.g., methane, nitrous oxide, and carbon dioxide) generated during milk production amount to approximately 1.4 billion metric tons, or 20%, of total emissions from the livestock industry [2]. Both the production and demand for milk have been increasing annually: in 2024, global milk production reached 940 million metric tons and is projected to rise to 1.146 billion metric tons by 2034 [3]. To meet rapidly increasing market demand, thousands of large-scale dairy farms have been established across China. Previous studies have demonstrated that intensive dairy production systems can reduce land requirements, improve land-use efficiency, and enhance feed utilization efficiency [4,5,6]. Nevertheless, the rapid expansion of industrial dairy farming represents a recent development in China. Unlike Western countries, where dairy consumption has deep historical and cultural foundations, dairy products have only recently become a mainstream component of the Chinese diet. Historically, milk consumption was largely restricted to pastoral regions in northern China, whereas dairy products were not traditionally consumed by the broader population. Furthermore, the high prevalence of lactose intolerance among the Chinese population constituted a persistent physiological barrier to widespread dairy consumption [7]. The convergence of rising living standards, accelerating urbanization, and technological innovations, particularly the development of lactose-free and fermented dairy products, has substantially increased consumer demand and, consequently, catalyzed the large-scale industrialization of the dairy sector in China. This model also provides new opportunities for centralized manure treatment, thereby reducing agricultural pollution [4,5,6,8]. In China, large-scale and intensive farming has become the default development model for dairy cattle breeding, accounting for 78% of all dairy farming in China in 2024 [9]. The GHG emission intensity and management level of intensive dairy farming play a critical role in determining the trajectory of low-carbon development within China’s dairy industry. Following China’s commitment to achieving carbon peaking and carbon neutrality, both newly established large-scale dairy farms and existing operations have increasingly been required to adopt low-carbon technologies and management practices, including biogas utilization, manure resource recycling, and precision feeding, either through facility retrofitting or replacement with more advanced production systems. Consequently, the current low-carbon performance of intensive dairy farming reflects an ongoing transition rather than an inherent characteristic of the industry. This transition has been progressively supported by an evolving policy and governance framework. At the national level, China has committed to achieving carbon peaking by 2030 and carbon neutrality by 2060 while enacting the Ecological Environment Code of the People’s Republic of China in 2026, thereby providing a legal framework for agricultural decarbonization [10]. Major policy instruments include the Implementation Plan for Agricultural and Rural Emission Reduction and Carbon Sequestration (2022), which establishes specific objectives for improving feed conversion efficiency and reducing GHG emission intensity per unit of agricultural output, and the Technical Guidance for Pollution and Carbon Reduction in Large-Scale Livestock Farms (2025), which recommends ten categories of mitigation technologies, including low-protein diet formulation, precision feeding, optimized manure management, and biogas recovery. In addition, the Regulations on the Prevention and Control of Pollution from Large-Scale Livestock Farms impose regulatory penalties, including financial fines and operational restrictions, on farms that fail to comply with waste treatment and environmental protection requirements. Nevertheless, the current regulatory framework remains predominantly incentive-oriented. Since 2017, the central government has invested approximately USD 4.6 billion in subsidies to promote manure resource utilization across 723 counties, whereas compliance with carbon-specific objectives continues to depend primarily on technical guidance and economic incentives rather than mandatory enforcement. Within this policy context, characterizing the GHG emission profiles of large-scale dairy farms is essential for informing targeted mitigation strategies and facilitating carbon reduction throughout the dairy industry.

Low-carbon development was not an original objective of dairy intensification in China. During the initial phase of industrial expansion, policy and industry priorities focused primarily on increasing milk production, improving production efficiency, and standardizing farm management. Recently, the national carbon-peaking and carbon neutrality agenda has redirected both policy and industrial development toward mitigation-oriented practices, including precision feeding, manure resource utilization, biogas recovery, renewable energy substitution, and related emission reduction technologies. North China is a useful study region because it encompasses several of the country’s principal large-scale dairy production areas. Dairy farms in this region are characterized by intensive management systems, mechanized production processes, centralized manure treatment, and close integration with national dairy-processing and cold-chain networks. Consequently, farm-level GHG accounting in North China can provide empirical evidence to support mitigation strategies applicable to China’s large-scale dairy production systems.

Life cycle assessment (LCA) and farm simulation models have established the principal methodological framework for quantifying GHG emissions from dairy production systems. Previous studies have defined system boundaries and functional units, identified major emission hotspots, and evaluated mitigation scenarios across diverse geographic regions and production systems [11,12,13]. These studies demonstrate that enteric fermentation, feed production, energy consumption, and manure management constitute the principal contributors to the carbon footprint of milk production. However, many previous studies have relied on cross-sectional surveys, aggregated regional statistics, or simulation-based approaches. Such methodologies are less capable of capturing interannual variation within individual commercial farms or of directly associating year-to-year changes in GHG emissions with corresponding changes in herd structure, dietary composition, energy consumption, and manure management practices. This knowledge gap constrains the ability to distinguish persistent emission hotspots from year-specific variability and to identify the management indicators most strongly associated with differences between high- and low-emission dairy farms.

The present study selected 22 large-scale dairy farms that were continuously monitored from 2021 to 2023. The herd sizes on the farms ranged from 1000 to 20,000 head. We used an LCA framework to calculate total carbon equivalent emissions and emission intensity. The objectives of this study are to (1) analyze the temporal variation characteristics of farm GHG emissions and identify the emission patterns during the development of the production system; (2) compare high- and low-GHG-emission (HE-LE) farms to identify the differences in resource efficiency, emission sources, and management practices; and (3) analyze the emission regulatory mechanisms in large-scale farms by assessing nutritional strategies and manure treatment.

## 2. Materials and Methods

### 2.1. Study Design

#### Study Design and Farm Classification

**Figure 1:**
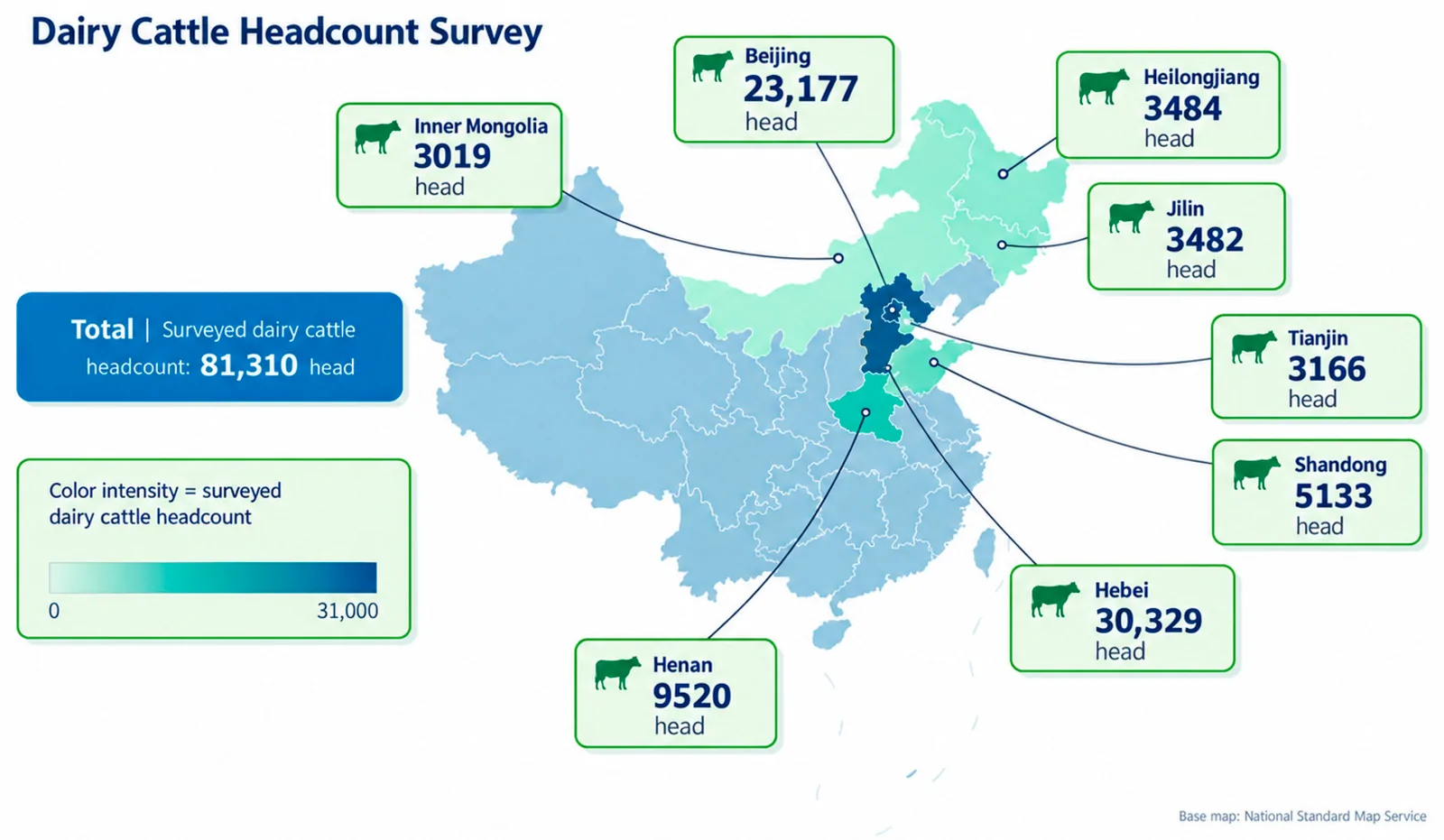
Distribution map of the surveyed pastures.

A longitudinal, multi-year repeated-measures design was used to measure greenhouse gas (GHG) emission intensity among large-scale intensive dairy farms in North China and evaluate its temporal trends, between-farm heterogeneity, and principal determinants. The observational sample included 22 commercial dairy farms, each with a herd size exceeding 1000 head and complete, uninterrupted production and management records throughout 2021–2023. Located in Beijing, Tianjin, Hebei, Henan, Inner Mongolia, Jilin, Shandong and Heilongjiang, the selected farms typified the region’s dominant large-scale, intensively managed production system (Figure 1).

To assess management attributes associated with farm-level differences in GHG emission intensity, farms were categorized annually into high-emission (HE) and low-emission (LE) groups. In each study year, farms within the highest quartile of GHG emission intensity per kg of fat- and protein-corrected milk (FPCM) formed the HE groups (*n* = 6), whereas those within the lowest quartile formed the LE group (*n* = 6). Group assignment was conducted separately for each year, thereby enabling individual farms to enter different emission categories during the 2021–2023 study period. Data collection and farm management adhered to standard operating procedures, and the animals received no experimental interventions.

### 2.2. Farm Selection and Data Collection

#### 2.2.1. Farm Selection Criteria

**Figure 2:**
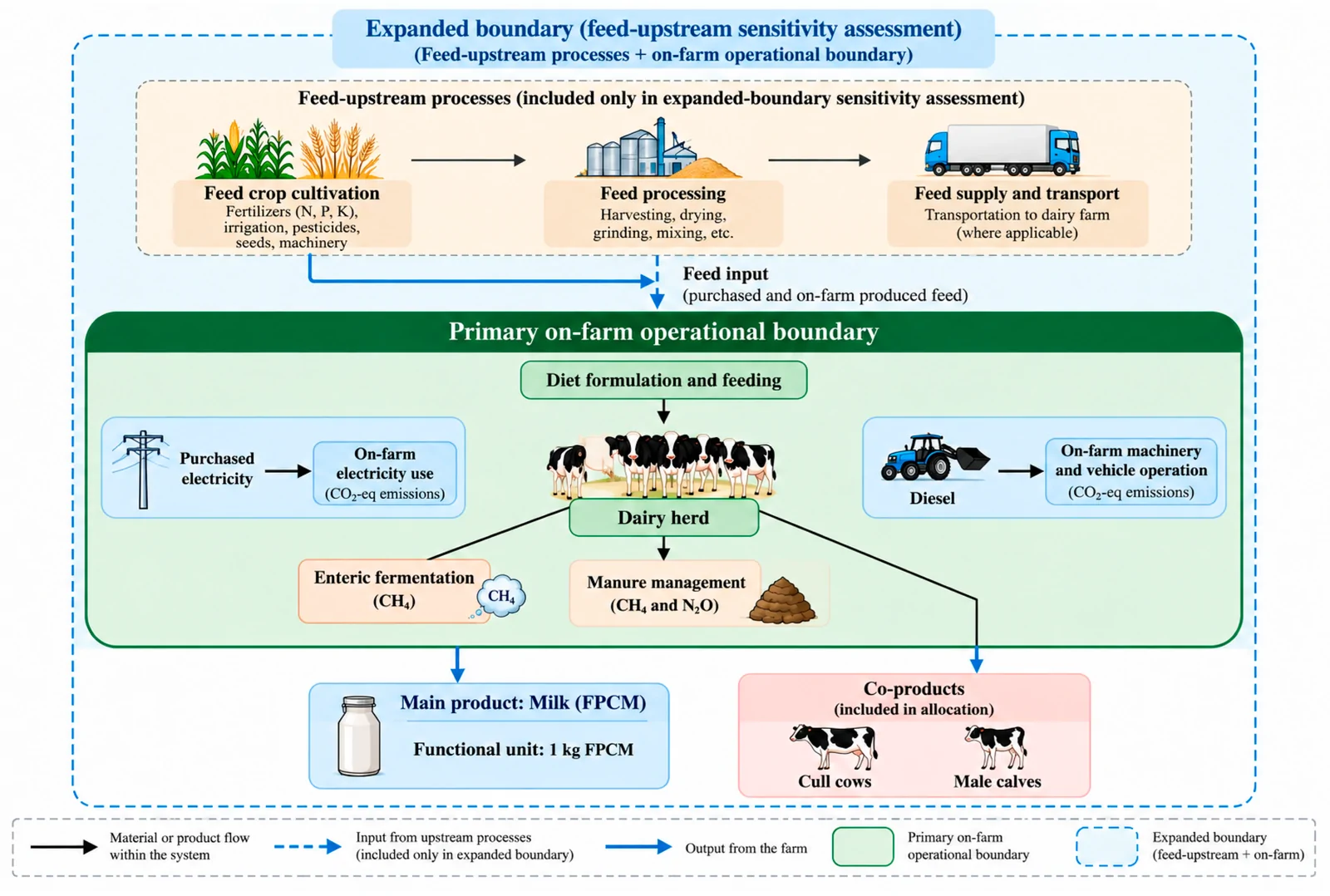
System boundaries, principal GHG emission sources, and associated product flows incorporated into the dairy farm assessment. The solid green boundary delineates the primary on-farm operational GHG inventory, encompassing diet formulation and feeding practices, enteric fermentation, manure management activities, purchased electricity consumption, and diesel use directly associated with dairy farm operations. The dashed blue boundary identifies the expanded system boundary employed for sensitivity analysis, additionally incorporating upstream feed production processes, including feed-crop cultivation, feed processing, and feed supply and transportation. Solid black arrows denote material or product flows within the dairy farm system, dashed blue arrows represent inputs originating from upstream processes, and solid blue arrows designate outputs generated by farm operations. Milk production, quantified as fat- and protein-corrected milk (FPCM), constitutes the principal product, with a functional unit defined as 1 kg FPCM; cull cows and male calves are identified as co-products accounted for in the allocation procedure. The 22 selected farms had a mean total herd inventory of 3950 ± 4040 head, of which lactating cows constituted 45.01 ± 1.96% (Table 1). The mean daily FPCM production per lactating cow was 35.03 ± 2.20 kg, and the mean lactating period was 320.45 ± 5.75 days per year, while the corresponding mean milk fat and protein contents were 3.81 ± 0.17% and 3.29 ± 0.06%, respectively.

Farms were selected based on the following criteria: (1) a total herd size exceeding 1000 head; (2) continuous production and management records spanning 2021 to 2023; (3) complete annual records of herd structure, milk production, diet composition, feed intake, energy consumption, and manure management; and (4) stable farm operation throughout the study period, with no substantial data omissions. The system boundary of GHG emission accounting is presented in Figure 2, covering enteric fermentation, energy consumption, and manure management.

**Table 1:** Characteristics of 22 large-scale dairy farms during 2021–2023.

Item	Mean	Maximum	Minimum	Standard Deviation
Herd structure:				
Total cows, head	3950	20954	1114	4040
Lactating cows, head	1738	8844	506	1751
Dry cows, head	250	1780	72	299
* Replacement heifer, head	1961	10804	527	2018
Female calves (0–6 months)	537	3033	145	558
Lactating cow ratio, %	45.01	49.29	41.06	1.96
Culled cows, head	750	4304	178	823
Male calf sales, head	653	3580	115	533
Milk production:				
Milk fat, %	3.81	4.20	3.57	0.17
Milk protein, %	3.29	3.46	3.17	0.06
FPCM production, kg/d	35.03	39.75	30.78	2.20
Diet composition, %:				
Forage	40.25	46.00	35.01	2.08
NDF	28.80	30.91	25.79	1.11
Starch	27.50	29.89	25.51	0.95
CP	16.56	18.38	15.90	0.47
DMI, kg/d	24.92	27.07	22.17	1.11
Feed efficiency	1.52	1.76	1.31	0.09
Energy consumption:				
Diesel, t	177.42	954.01	29.40	182.87
Electricity, MWh	4057	25670	703	4810

Abbreviations: DMI, dry matter intake; FPCM, fat- and protein-corrected milk. * Replacement heifers encompass female calves aged 0–6 months; female calves are presented separately as a distinct physiological subgroup and must not be counted twice when determining the total herd population.

#### 2.2.2. Data Sources and Collection Procedures

Primary data were obtained from standardized on-farm production and management recording systems. Each farm maintained digital records to ensure data accuracy, traceability, and temporal continuity. The following data categories were compiled annually:

(a) Herd structure and productivity data: Annual records comprised total herd size; numbers of lactating cows, dry cows, replacement heifers and calves; milk yield; milk fat content; milk protein content; sales of male calves; and culled cow numbers. According to the farm records, replacement heifers comprised the entire female replacement population before first calving, thereby encompassing female calves. To differentiate among physiological developmental stages, female calves aged 0–6 months were further classified as a distinct subgroup within the replacement-heifer population. Female calves were raised on the farms and retained for subsequent herd replacement, except for those culled due to health conditions or developmental abnormalities. As female calves remained within the herd rather than being sold, they were excluded from the co-product allocation calculations. In contrast, male calves were marketed by the farms and consequently accounted for as co-products in the economic allocation procedure. Artificial insemination was routinely used across the surveyed farms, with sexed semen used for replacement heifers and conventional semen used for mature cows.

(b) Feed and nutrition data: Annual total feeding amounts for purchased feeds (e.g., roughage and concentrates) were determined based on farm feed procurement contracts and feeding records. Feed nutritional composition data were obtained through sampling conducted by the nutrition departments for each farm, and the contents of starch, CP, and NDF were determined according to AOAC [14] standard methods.

(c) Manure management data: Manure storage methods, storage duration, and final utilization pathways were recorded.

(d) Energy consumption data: Annual diesel consumption (e.g., farm machinery operations) and electricity consumption (e.g., lighting, ventilation, and milking equipment) were verified using farm energy bills and equipment operation logs.

### 2.3. GHG Accounting Framework and System Boundary

The GHG accounting framework was developed with reference to ISO 14040 and ISO 14044 international standards [15,16] and the Livestock Environmental Assessment and Performance Guidelines of the Food and Agriculture Organization [17,18]. The primary analysis was designed as a farm-level operational GHG inventory rather than a complete cradle-to-farm-gate product carbon footprint.

The primary GHG assessment employed an on-farm operational system boundary, established with reference to ISO 14064-1:2018 [19] and the IPCC 2019 Refinement [20]. This system boundary encompassed GHG emissions directly generated by or attributable to dairy farm activities, specifically including (1) methane (CH_4_) emissions arising from enteric fermentation, (2) CH_4_ and nitrous oxide (N_2_O) emissions originating from manure management, and (3) carbon dioxide (CO_2_) emissions associated with electricity and fuel consumption during on-farm operations. Upstream GHG emissions arising from feed-crop cultivation, fertilizer manufacturing and application, crop harvesting, feed processing, and off-farm feed transportation were excluded from the principal operational inventory. Similarly, downstream activities occurring after milk departed the farm, encompassing milk transportation, processing, distribution, and retail, were excluded from the primary system boundary. This operational system boundary was consistently applied across all 22 dairy farms and served as the basis for interannual comparisons, comparisons between high- and low-emission farms, correlation analyses, and identification of key farm management factors influencing GHG emissions.

To assess the effects of expanding the system boundary on GHG emission intensity expressed per unit of fat- and protein-corrected milk (FPCM), a supplementary sensitivity analysis incorporating upstream feed production emissions was performed. This additional assessment accounted for upstream GHG emissions associated with feed production by integrating farm-specific feed consumption records with emission factors corresponding to individual feed ingredients. The expanded boundary analysis was conducted to determine the additional GHG emission burden attributable to upstream feed production, while retaining the primary on-farm operational inventory as the basis for evaluating farm-level emission characteristics and management-related determinants.

The primary operational and expanded system boundaries are depicted separately in Figure 2. The primary operational boundary encompasses GHG emissions directly attributable to dairy-farm activities, whereas the expanded boundary employed in the sensitivity analysis additionally accounts for emissions arising from upstream feed production processes.

### 2.4. Expanded-Boundary Feed-Upstream Sensitivity Assessment

The primary GHG inventory established in this study employed an on-farm operational system boundary, which remained unchanged throughout all interannual comparisons, high- versus low-emission (HE–LE) comparisons, correlation analyses, and assessments of farm management drivers. To assess the effects of expanding the system boundary on GHG emission estimates, a supplementary sensitivity analysis incorporating upstream feed production emissions was performed using annual feed consumption records collected from all 22 dairy farms between 2021 and 2023.

For each individual feed ingredient, annual consumption expressed on a fresh-matter basis was converted into the corresponding dry-matter quantity by applying ingredient-specific dry-matter fractions. The associated upstream GHG emissions were subsequently calculated as follows:

*Feed GHG* = *feed consumption* × *dry* − *matter fraction* × *ingredient* − *specific emission factor* (1)

Ingredient-specific GHG emission factors were applied to different feed categories, including forage crops, cereal grains, protein-rich feeds, agro-industrial by-products, compound feeds, mineral supplements, and feed additives. Whenever supplier-specific primary emission data on feed cultivation practices, fertilizer inputs, processing technologies, and transportation distances were unavailable, proxy emission factors derived from the published literature were used as alternative estimates. To evaluate the uncertainty associated with the use of secondary emission factors in the expanded system boundary, additional sensitivity scenarios were developed by applying a uniform ±25% variation to the baseline feed-upstream emission factors. Specifically, the low-emission scenario was calculated by multiplying each baseline emission factor by 0.75, whereas the high-emission scenario was obtained by multiplying each baseline emission factor by 1.25. This ±25% range was adopted as an assumed sensitivity interval to assess the responsiveness of the results to variations in feed-upstream emission factors, rather than as a statistically derived uncertainty range.

All feed ingredients were purchased from external suppliers. Upstream GHG emissions associated with feed cultivation, processing, and transportation were estimated using ingredient-specific or literature-based proxy emission factors.

The upstream GHG emission intensity attributable to feed production was determined using the identical total fat- and protein-corrected milk (FPCM) denominator employed in the production-weighted operational GHG inventory, as expressed below:

(2) $$\mathrm{CF\_feed}\;(\mathrm{kg}\;{\mathrm{CO}}_{2}\mathrm{eq/kg})\;=\frac{\mathrm{total}\;\mathrm{feed}\;\mathrm{upstream}\;\mathrm{GHG}\;\mathrm{emissions}\;(\mathrm{kg}\;{\mathrm{CO}}_{2}\mathrm{eq/kg})\;}{\;\mathrm{total}\;\mathrm{FPCM}\;\mathrm{production}\;(\mathrm{kg})}$$

where *CF_feed* is the carbon footprint from upstream feed production sectors.

The expanded-boundary GHG intensity was calculated as

*CF*_*expanded* (kg CO_2_eq/kg) = *CF_on_farm* (kg CO_2_eq/kg) + *CF*_*feed* (kg CO_2_eq/kg) (3)

where *CF_expanded* is the carbon footprint from upstream feed production and on-farm sectors.

This supplementary expanded-boundary assessment was performed specifically to evaluate the sensitivity of GHG emission estimates to system boundary selection, while the original on-farm operational inventory remained unchanged and continued to serve as the basis for high- versus low-emission (HE–LE) farm comparisons and analyses of farm management drivers.

### 2.5. Functional Unit and Co-Product Allocation

#### 2.5.1. Functional Unit

Fat- and protein-corrected milk (FPCM) was selected as the functional unit to facilitate standardized comparisons of GHG emission intensity among farms and across years. FPCM was measured using the internationally recognized formula [21]:

*FPCM* (kg) = *Milk Yield* (kg) × (0.1226 × *Fat*% + 0.0776 × *Protein*% + 0.2534) (4)

#### 2.5.2. Co-Product Allocation

Dairy production systems generate milk as the primary product, whereas meat from culled cows and male calves represents economically valuable co-products. In this study, the functional unit was defined as 1 kg of fat- and protein-corrected milk (FPCM). Because milk and meat are expressed in different physical units and cannot be directly compared on a mass basis, economic allocation was applied to distribute total GHG emissions according to their relative economic values during the same production period. The economic value of milk was calculated by multiplying the quantity of saleable milk by its farm-specific price, whereas the economic value of meat co-products was calculated from the numbers of culled cows and male calves sold and their corresponding sale prices. The economic allocation fraction assigned to milk was calculated as follows:

(5) $$\mathrm{Allocation}\;\mathrm{of}\;\mathrm{Milk}\;=\frac{\mathrm{Revenue}\;\mathrm{of}\;\mathrm{milk}}{\mathrm{Revenue}\;\mathrm{of}\;\mathrm{milk}+\mathrm{Revenue}\;\mathrm{of}\;\mathrm{culled}\;\mathrm{cows}+\mathrm{Revenue}\;\mathrm{of}\;\mathrm{calves}\;\mathrm{sale}}$$

The sale prices of culled cows and male calves were set at constant prices of 12,500 CNY and 700 CNY per head, respectively. The average price per kilogram of milk across the 22 farms was 3.88 CNY, with slight variations among farms. These variations mainly arose from differences in regional base prices, purchasers, and premiums or subsidies for milk fat, milk protein, and transportation provided by dairy companies. The specific price per kilogram of milk can be found in the data package in the bibliography.

### 2.6. GHG Emission Accounting Model and Emission Factors

#### 2.6.1. Methane Emissions from Enteric Fermentation

Enteric methane (CH4) emissions were quantified using the tier 2 estimation methodology outlined in the IPCC (2019) guidelines [20]. For each farm, the methane conversion factor (Ym) was determined from the gross energy (GE) content of the total mixed ration (TMR) provided. The animal-specific Ym values were assigned as 5.7% for lactating cows and 6.3% for dry cows and heifers, consistent with the herd composition IPCC [20].

#### 2.6.2. Methane and Nitrous Oxide Emissions from Manure Management

The IPCC (2019) Tier 2 method [20] was applied to estimate manure-derived CH_4_ emissions from volatile solids (VSs) excretion, maximum methane-producing capacity (B_0_), methane conversion factors (MCFs), and the manure proportions allocated to individual management systems (Table 2). The IPCC (2019) Tier 2 method [20] was also used to estimate direct and indirect manure management N_2_O emissions from manure nitrogen (N) excretion, direct N_2_O emission factors (EF_3_), and N volatilization fractions (Frac) (Table 2).

#### 2.6.3. Carbon Dioxide Emissions from Energy Use

Electricity: The annual electricity consumption for each farm was multiplied by the official province-specific grid electricity CO_2_ emission factor to estimate CO_2_ emissions. The emission factors were obtained from the 2022 Electricity CO_2_ Emission Factors released by the Ministry of Ecology and Environment and the National Bureau of Statistics (Table 3) [26]. The electricity emission factors established for 2022 were consistently applied throughout the entire study period to maintain a standardized coefficient basis for evaluating variations in electricity consumption among dairy farms and across different years. Consequently, the resulting electricity-related GHG emission estimates should be interpreted as reflecting differences in electricity consumption under a fixed emission factor framework, rather than accounting for annual fluctuations in the electricity generation mix of the power grid.

Diesel: Annual diesel consumption was multiplied by the IPCC-recommended emission factor of 3.096 kg CO_2_ eq kg^−1^ diesel consumed to estimate CO_2_ emissions [20].

### 2.7. Model Validation and Uncertainty Analysis

The following analyses were undertaken to evaluate the effects of input parameters on the resulting estimates and quantify the aggregate uncertainty in the calculated GHG footprints.

#### 2.7.1. Sensitivity Analysis

Each key model parameter, comprising dietary gross energy, methane conversion factors (Ym), and manure management system factors (MCF, EF_3_, and Frac), was perturbed individually by ±10% relative to its baseline value. For each perturbation, the percentage response of the GHG footprint per kg FPCM was documented to determine which parameters had the greatest influence.

#### 2.7.2. Uncertainty Propagation Analysis

Monte Carlo uncertainty simulation was implemented in the SimaPro 9.0 software (PRé Sustainability B.V., The Netherlands). Probability distributions representing the principal input parameters were specified, after which the model was run for 10,000 iterations to produce a distribution of plausible GHG footprint outcomes. Simulation outputs were used to derive the standard deviation and 95% confidence intervals of the final GHG footprint estimates. Reported emission intensities are expressed as mean estimates with their corresponding 95% confidence intervals.

For the expanded-boundary feed-upstream assessment, a scenario-based sensitivity analysis was additionally conducted by applying a uniform ±25% variation to the baseline emission factors of all feed ingredients. The low- and high-emission scenarios were calculated using 75% and 125% of the corresponding baseline emission factors, respectively. This range was adopted as an assumed sensitivity scenario rather than a literature-derived uncertainty interval.

### 2.8. Statistical Analysis

All statistical analyses were conducted using the SAS software, version 9.4 (SAS Institute Inc., Cary, NC, USA) [27].

#### 2.8.1. Interannual Comparisons

Within-farm differences in GHG footprint across the three study years were analyzed using repeated-measures analysis of variance (ANOVA) with the PROC GLM procedure. Correlation arising from repeated observations of the same experimental unit (i.e., farm) was incorporated into this model. The Greenhouse–Geisser correction was implemented whenever the sphericity assumption was not satisfied.

#### 2.8.2. Between-Group Comparisons (HE vs. LE)

Differences between high- and low-emission farm groups in GHG footprint and management indicators were assessed using independent-samples *t*-tests (PROC TTEST) when the normality and homogeneity-of-variance assumptions were satisfied. If either assumption was not satisfied, the nonparametric Mann–Whitney U test was applied.

### 2.9. Correlation Analysis

**Figure 3:**
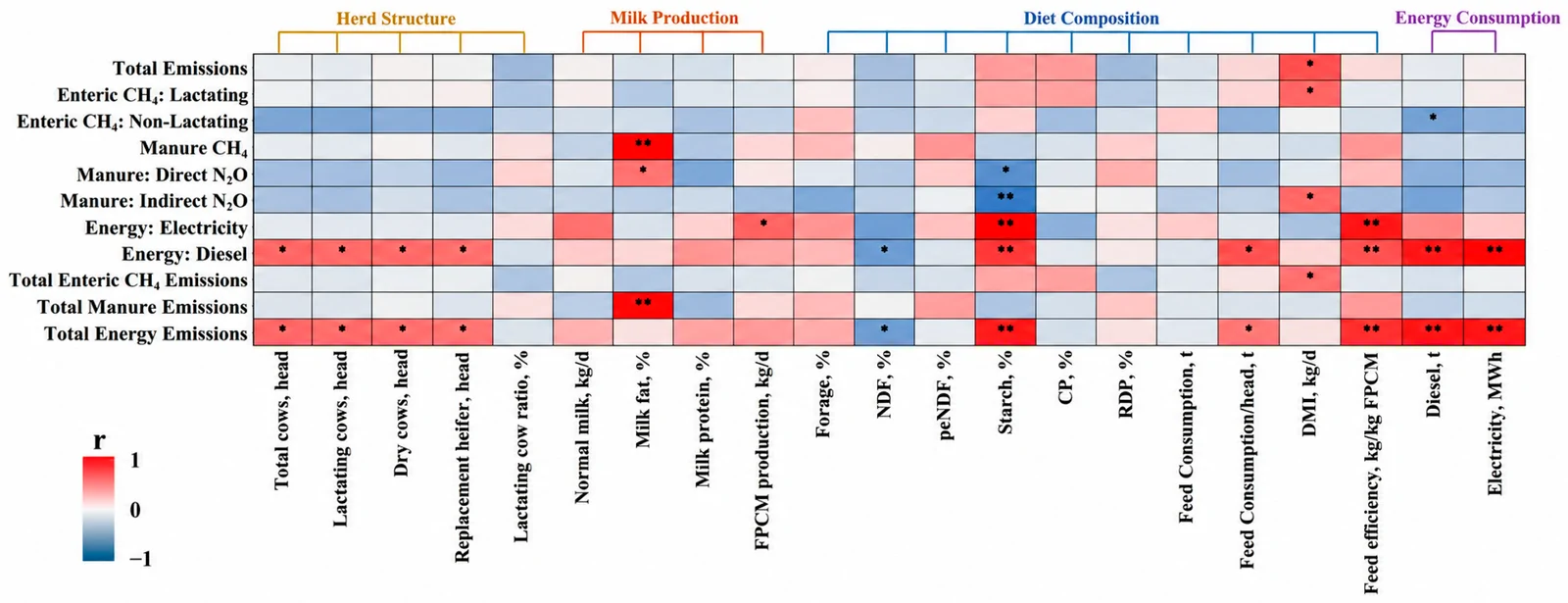
Correlation analysis between farm production structure, performance, and GHG footprint. “*” indicates *p* < 0.05; “**” indicates *p* < 0.01. Abbreviations: FPCM, fat- and protein-corrected milk; peNDF, physically effective neutral detergent fiber; RDP, rumen degradable protein; DMI, dry matter intake.

Pearson correlation coefficients were estimated to characterize associations among farm production structure, performance indicators, and GHG footprint components and were displayed in a correlation matrix (Figure 3), with significance levels indicated (*p* < 0.05; * *p* < 0.01).

The results are summarized as means ± standard error of the mean (SEM). Statistical significance was established at α = 0.05.

## 3. Results

### 3.1. Analysis of Farm Management for 2021–2023

This study analyzed annual variations in farm production structure and performance indicators from January 2021 to December 2023 (Table 4). The total herd size increased over the three-year period, although this change was not statistically significant (*p* = 0.055). The number of lactating cows increased significantly (*p* < 0.05) from 1692 in 2021 to 1867 in 2023. The number of replacement heifers aged 0–24 months also increased, although this change was not statistically significant (*p* > 0.05). According to the farm records, the replacement-heifer category encompassed all female animals retained for herd replacement within the 0–24-month age range. To differentiate physiological developmental stages, female calves aged 0–6 months were also identified in Table 4 as a distinct subgroup within the replacement-heifer population and were, therefore, excluded from separate enumeration when determining the total herd population. Notably, the proportion of lactating cows declined significantly (*p* < 0.05), from 46.0% in 2021 to 44.3% in 2023, suggesting an increase in the relative size of the non-productive herd component within the overall herd structure. The yield of FPCM increased significantly (*p* < 0.05), from 34.35 kg/d to 35.78 kg/d during the study period. Milk production without component correction did not change (*p* = 0.055). Feed conversion efficiency increased significantly (*p* < 0.001), improving from 1.45 to 1.58 kg FPCM/kg DMI. The milk fat percentage was significantly higher in 2021 and 2023 compared with 2022. Milk fat percentage was higher in 2023 than in 2021 (*p* < 0.05). Milk protein percentage peaked in 2022 and was significantly higher than in 2021 and 2023 (*p* < 0.05). The forage proportion of the diet increased significantly over the study period (*p* < 0.05), and dietary starch content also increased significantly (*p* < 0.05), from 26.69% in 2021 to 28.23% in 2023. Conversely, dietary crude protein and rumen degradable protein (RDP) decreased significantly in 2023 (*p* < 0.05). Total NDF did not change (*p* > 0.05), while DMI increased significantly in 2023 (*p* < 0.05). Differences were observed in diesel and electricity consumption (*p* < 0.05), with average annual diesel consumption increasing from 138.55 tons in 2021 to 213.07 tons in 2023, and annual electricity consumption increasing from 3.294 million kWh to 4.657 million kWh. Manure management practices remained consistent across all farms throughout the study period (Table 5). Anaerobic digestion was the predominant treatment method, accounting for 69.8% of total manure handled annually. Off-site field application accounted for 21.5%. The use of oxidation ponds and 3-month under-barn storage was limited.

### 3.2. Analysis of GHG Footprint Contribution and Emissions by Source for 2021–2023

**Figure 4:**
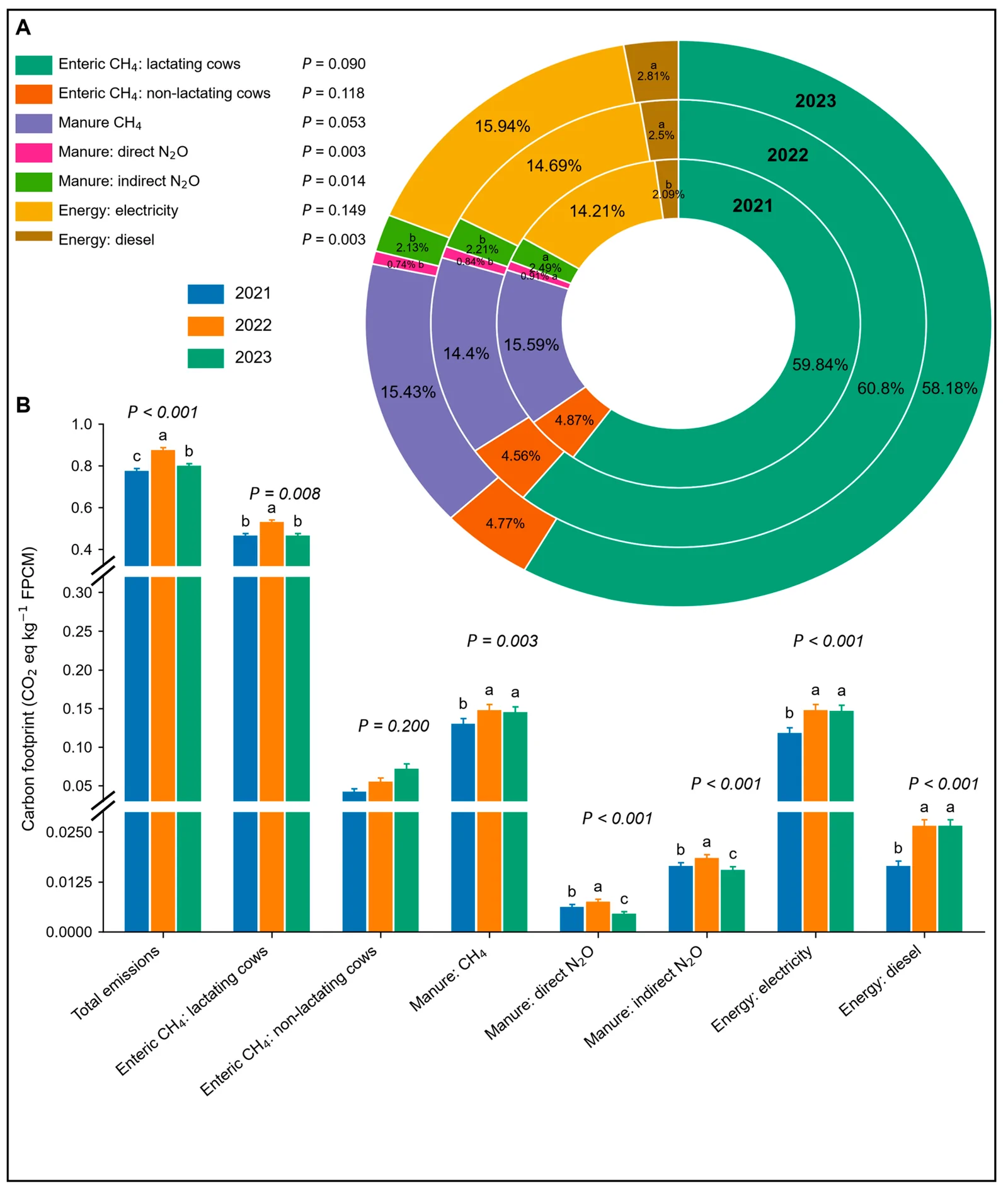
Analysis of GHG footprint contribution and emissions by source for 2021–2023. (**A**) Donut chart showing the percentage contribution of each emission source to total GHG footprint. (**B**) Bar chart illustrating absolute emissions (CO_2_ eq kg^−1^ FPCM) from each source. Different lowercase letters (a–c) indicate significant differences among means (*p* < 0.05), whereas means sharing the same letter are not significantly different.

The results are presented in Figure 4. While the overall emission structure remained stable across years, there were significant interannual variations in the contribution rates of certain sources. Specifically, the percentage contributions to total carbon equivalent emissions were methane from enteric fermentation in lactating cows (59.6%), methane from manure management (15.1%), carbon dioxide emissions from electricity consumption (14.9%), methane from enteric fermentation in non-lactating cattle (5.3%), carbon dioxide emissions from diesel consumption (2.4%), indirect nitrous oxide emissions from manure (2.2%), and direct nitrous oxide emissions from manure (0.8%). Enteric methane from all cattle (lactating and non-lactating combined) constituted the largest emission source (64.9%), representing the main component of the farm-level GHG footprint. Analysis of the 2021–2023 data revealed that the proportional contributions of direct and indirect manure-derived N_2_O emissions were significantly greater in 2021 than in 2022 and 2023 (*p* < 0.05). In contrast, the contribution of direct CO_2_ emissions was significantly higher in 2022 and 2023 than in 2021 (*p* < 0.05). No significant interannual differences were observed in the proportional contributions of total enteric CH_4_ emissions (from lactating and non-lactating cattle), manure-derived CH_4_ emissions, or electricity-related CO_2_ emissions (*p* > 0.05). To quantify GHG emissions from farm production systems, we further calculated the GHG footprint per kilogram of FPCM for farm operations (Figure 4B). The GHG footprint was highest in 2022 (0.86 kg CO_2_ eq kg^−1^ FPCM) and lowest in 2021 (0.77 kg CO_2_ eq kg^−1^ FPCM), with a three-year average of 0.86 kg CO_2_ eq kg^−1^ FPCM. Analysis of absolute emission intensity by source showed that enteric methane emissions from lactating cows in 2022 were significantly higher than in 2021 and 2023 (*p* < 0.05). Moreover, emissions from manure methane, electricity, and diesel were significantly higher in 2022 and 2023 than in 2021 (*p* < 0.05). Conversely, emissions from both direct and indirect manure nitrous oxide in 2021 were significantly lower than in 2022 but higher than in 2023 (*p* < 0.05).

### 3.3. GHG Footprint Analysis of High- and Low-Greenhouse-Gas-Emission Farms

**Figure 5:**
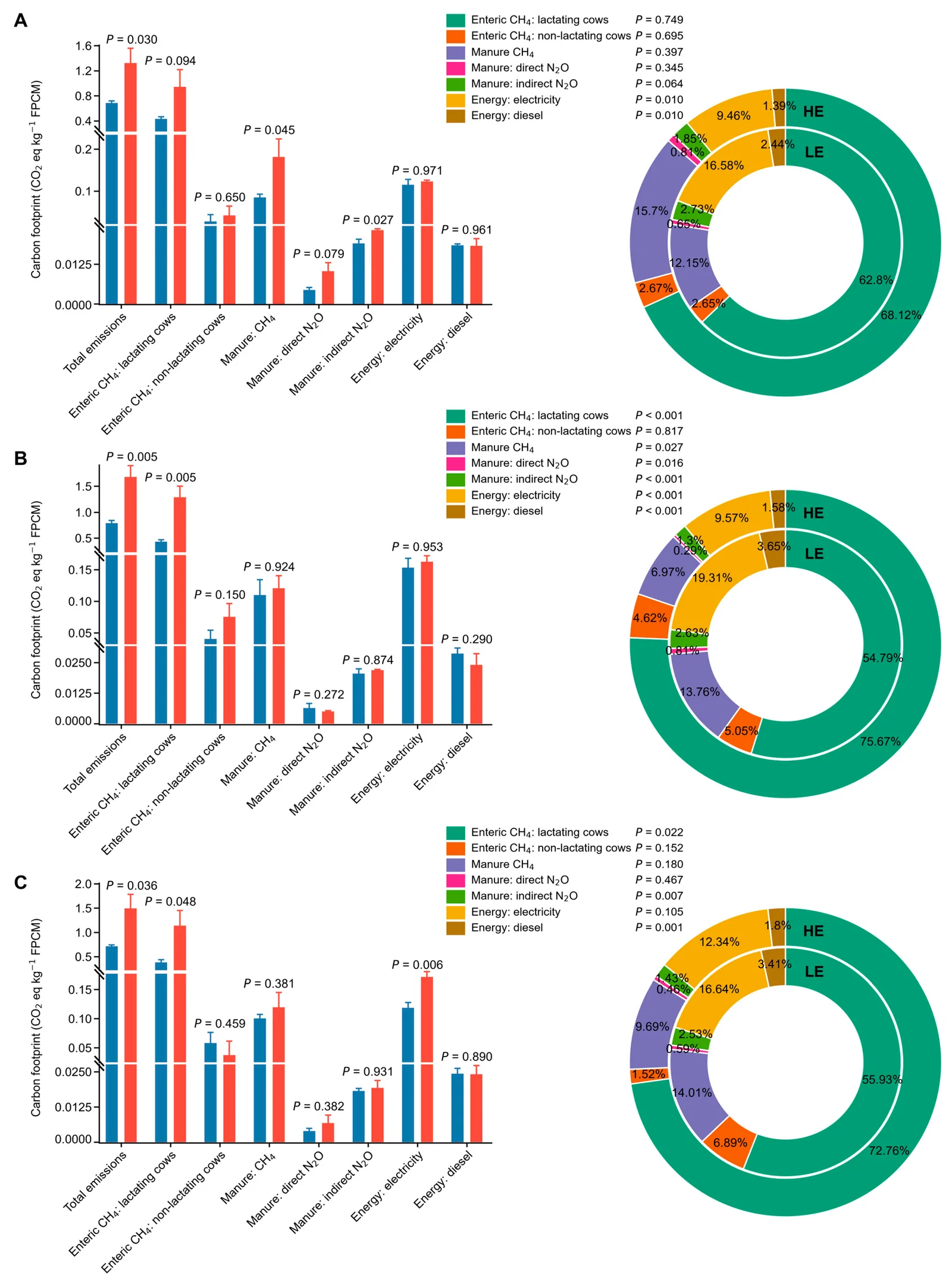
Analysis of carbon footprint contribution and emissions for high- and low-GHG-emission farms for 2021–2023. (**A**–**C**) correspond to the analyses for 2021, 2022, and 2023, respectively. HE: high-greenhouse-gas-emissions group; LE: low-greenhouse-gas-emissions group.

From 2021 to 2023, differences were observed in both the proportional structure and absolute magnitude of the GHG footprint between HE and LE farms (Figure 5A, Figure 5B, and Figure 5C correspond to each year, respectively). In 2021, total carbon equivalent emissions per kg FPCM, as well as emissions from manure methane, indirect manure nitrous oxide, and the proportional contributions from electricity and diesel, were significantly higher in HE farms than in LE farms (*p* < 0.05). However, emissions from enteric methane in lactating cows (*p* = 0.094) and direct manure nitrous oxide (*p* = 0.079) did not change. In 2022, total carbon equivalent emissions per kg FPCM and the absolute amount and proportional contribution of enteric methane from lactating cows were significantly higher in HE farms (*p* < 0.05). Conversely, owing to the increased dominance of enteric methane, the proportional contributions of emissions from manure methane, manure nitrous oxide, and energy consumption (electricity and diesel) were significantly lower in HE farms than in LE farms (*p* < 0.05). Similarly, in 2023, total carbon equivalent emissions per kg FPCM and the absolute amount and proportional contribution of enteric methane from lactating cows were also significantly higher in HE farms (*p* < 0.05). Furthermore, HE farms had a significantly higher proportional contribution from electricity emissions (*p* < 0.05), along with significantly lower proportional contributions from indirect manure nitrous oxide and diesel emissions (*p* < 0.05).

### 3.4. Analysis of Farm Management in High- and Low-GHG-Emission Farms

In 2021, the total herd size was greater at LE farms than at HE farms (Table 6). However, the herd size at HE farms increased from 2022 to 2023, resulting in total herd size on HE farms surpassing that of LE farms by 2023. The trends in the number of lactating cows, dry cows, and replacement heifers followed a similar pattern: LE farms had larger herd sizes in 2021, whereas HE farms reached larger herd sizes by 2023. LE farms maintained a higher percentage of lactating cows than HE farms across all three years. In contrast with herd structure trends, differences were observed in lactation performance. In 2021, the average daily yields of both standard milk and FPCM were lower at LE farms than at HE farms. However, in 2022 and 2023, milk production at LE farms exceeded that at HE farms. Milk fat percentage and milk protein percentage did not differ between the two farm types, but interannual patterns were inconsistent. Differences in dietary composition (including forage, NDF, and starch) between the two farm types were not significant. DMI was marginally higher at LE farms than at HE farms over the three-year period. In terms of total feed consumption, LE farms substantially exceeded HE farms in 2021 but, by 2023, HE farms had surpassed LE farms. Conversely, feed consumption per cow was consistently higher at HE farms than at LE farms across all three years. Feed efficiency varied across years: it was higher at HE farms in 2021, but LE farms achieved equal or higher efficiency in 2022 and 2023. In contrast, diesel use was higher at LE farms than at HE farms during 2021 and 2022, but HE farms showed a significant increase in diesel consumption in 2023, and it was higher than at LE farms. Except for 2021, electricity consumption was higher at HE farms than at LE farms.

Anaerobic digestion was the primary manure management method for both farm types (Table 7). However, its proportion varied both between years and between groups. In 2021, anaerobic digestion accounted for 95% of manure management in LE farms, compared with only 52.7% in HE farms. By 2022, this proportion increased to 95% in HE farms and decreased to 76.6% in LE farms. In 2023, the proportion in LE farms was 92.5%, whereas in HE farms, anaerobic digestion decreased to 84.4%, with both groups maintaining a high overall adoption rate. The use of oxidation ponds was low throughout the study period, and proportions were similar between the two groups each year. The 3-month under-barn storage method was utilized only in HE farms in 2021 and was not adopted by either group in the subsequent two years. The proportion of off-site field application showed considerable interannual variation. In 2021, under-barn storage was substantially higher in HE farms than in LE farms. In 2021, off-site field application was more common in HE farms than in LE farms. In 2022, the proportion of off-site field application increased to 17.5% in LE farms but decreased to 0.25% in HE farms. By 2023, it increased to 11.6% in HE farms and decreased to 0.8% in LE farms.

### 3.5. Correlation Analysis Between Herd Production Structure, Performance, and GHG Footprint Components

As shown in Figure 2, indicators of herd production structure, milk production, dietary composition, energy consumption, and various components of the GHG footprint were correlated. Regarding herd structure, total herd size and the number of lactating cows, dry cows, and replacement heifers were all positively correlated with total energy-related emissions and, in particular, diesel emissions (*p* < 0.05). This suggests that farm expansion may lead to increased energy consumption and associated GHG emissions. In contrast, the proportion of lactating cows was not correlated with emission levels (*p* > 0.05). Regarding milk production indicators, milk fat percentage was positively correlated with total manure emissions, manure methane emissions, and indirect manure nitrous oxide emissions (*p* < 0.05). The yield of FPCM was positively correlated with emissions from electricity consumption (*p* < 0.05). Regarding dietary and feed-related variables, the dietary starch proportion was negatively correlated with both direct and indirect manure nitrous oxide emissions (*p* < 0.05) but positively correlated with total energy-related emissions and their subcomponents (*p* < 0.05), namely, diesel and electricity emissions. Feed consumption per cow was positively correlated with total energy and diesel emissions (*p* < 0.05). Dry matter intake (DMI) was positively correlated with total carbon emissions, total enteric methane emissions, enteric methane from lactating cows, and indirect manure nitrous oxide emissions (*p* < 0.05). Feed efficiency was positively correlated with total energy emissions and, in particular, diesel and electricity emissions (*p* < 0.05). In terms of energy consumption, both diesel and electricity consumption were positively correlated with their respective energy-related emissions (*p* < 0.05). Additionally, diesel consumption was negatively correlated with enteric methane emissions from non-lactating cattle (*p* < 0.05).

### 3.6. Expanded-Boundary Sensitivity Analysis of GHG Intensity

The production-weighted GHG emission intensity calculated under the primary on-farm operational boundary was 0.860 kg CO_2_-eq kg^−1^ FPCM, consistent with the value presented in the principal assessment. The additional upstream GHG emissions attributable to feed production contributed 0.427 kg CO_2_-eq kg^−1^ FPCM. Incorporating this upstream emission component increased the overall GHG emission intensity under the expanded system boundary to 1.287 kg CO_2_-eq kg^−1^ FPCM, corresponding to a 49.7% increase compared with the primary operational inventory (Table 8).

Upstream emissions arising from feed production constituted 36.7% of the total GHG burden estimated under the expanded system boundary. When the lower- and upper-bound ingredient-specific emission factors were applied in the sensitivity scenarios, the resulting expanded-boundary GHG emission intensity varied between 1.193 and 1.478 kg CO_2_-eq kg^−1^ FPCM.

The findings obtained under the expanded system boundary are presented as a supplementary sensitivity assessment to complement the primary operational inventory. The previously reported analyses of interannual variability, comparisons between high- and low-emission (HE–LE) farms, correlation analyses, and identification of farm management drivers continued to rely exclusively on the predefined on-farm operational system boundary.

## 4. Discussion

In the present study, we analyzed the GHG emission characteristics, management practices, and differences between high- and low-emission farms across 22 large-scale dairy farms from 2021 to 2023 in North China. Within the predefined on-farm operational system boundary, enteric methane emissions constituted 64.9% of the total quantified GHG emissions, exceeding the approximately 35–56% contribution documented in several previous dairy life cycle assessment (LCA) studies employing cradle-to-farm-gate system boundaries [28,29,30,31,32,33,34]. However, when feed-upstream emissions were included in the expanded-boundary assessment, the contribution of enteric methane decreased to 43.4%, consistent with the range reported in these studies. This finding highlights the influence of system-boundary selection on the relative contributions of GHG emission sources. Consistent with the importance of upstream feed-related emissions, feed production has been reported to account for approximately 26% of total GHG emissions in US dairy production systems [27,31,34].

The production-weighted mean GHG emission intensity of 0.86 kg CO_2_-eq kg^−1^ FPCM presented in the primary assessment reflects emissions generated within the predefined on-farm operational system boundary, encompassing enteric fermentation, manure management, purchased electricity consumption, and fuel use directly associated with dairy-farm activities. This standardized operational boundary was consistently applied to all 22 dairy farms and consequently served as the methodological basis for interannual comparisons, high- versus low-emission (HE–LE) farm comparisons, correlation analyses, and identification of management-related factors influencing GHG emissions.

Nevertheless, the GHG emission intensity estimated under this operational boundary should not be regarded as a cradle-to-farm-gate carbon footprint of milk production, as upstream emissions attributable to feed production were excluded from the primary inventory. This methodological distinction is relevant when evaluating the respective contributions of electricity consumption and diesel use to overall GHG emissions. The diesel emission category within the operational inventory accounts exclusively for fuel consumed directly during dairy farm activities. Accordingly, this category excludes diesel consumption and other energy inputs associated with crop cultivation, harvesting, feed processing, and supply chain operations involving externally procured feed ingredients. Consequently, the relatively limited contribution of direct diesel consumption should not be interpreted as evidence that agricultural field operations predominantly relied on electrification; instead, a considerable proportion of field-related energy consumption occurred beyond the predefined on-farm operational system boundary.

To quantify the magnitude of these excluded upstream GHG emissions, an additional expanded-boundary sensitivity assessment was conducted using farm-specific feed consumption records collected over three consecutive years. All feed ingredients were purchased from external suppliers, and their upstream GHG emissions were estimated using ingredient-specific or literature-based proxy emission factors.

By applying ingredient-specific upstream GHG emission factors, the additional emission burden attributable to feed production was quantified as 0.427 kg CO_2_-eq kg^−1^ FPCM. Incorporating this upstream contribution into the original operational GHG emission intensity of 0.860 kg CO_2_-eq kg^−1^ FPCM yielded an expanded-boundary estimate of 1.287 kg CO_2_-eq kg^−1^ FPCM. Accordingly, accounting for upstream feed production emissions increased the estimated GHG burden by 49.7%, with these upstream emissions contributing 33.2% of the total GHG emissions calculated under the expanded system boundary. These findings demonstrate that the selection of system boundaries substantially influences the absolute GHG emission intensity reported per kg FPCM.

Nevertheless, the expanded-boundary calculation fulfills an analytical objective distinct from that of the primary on-farm operational inventory. The primary assessment was intended to characterize emission sources and identify management-related determinants directly attributable to dairy farm activities, whereas the expanded-boundary analysis was designed to quantify the additional upstream GHG burden associated with feed production. Accordingly, these two assessment approaches should be regarded as complementary analytical perspectives rather than directly interchangeable accounting frameworks. Notably, the supplementary calculation of upstream feed production emissions did not alter the original HE–LE farm classification or supersede the interannual comparisons, correlation analyses, and assessments of farm management drivers conducted under the primary operational boundary.

Several sources of uncertainty remain associated with the expanded-boundary assessment of upstream feed production emissions. Although ingredient-level feed consumption records were available for all investigated farms, supplier-specific data concerning crop origin, fertilizer application practices, agricultural machinery use, feed-processing technologies, and transportation distances were not consistently obtainable. Consequently, ingredient-specific GHG emission factors derived from the published literature were applied, with proxy emission factors additionally required for certain commercial concentrates, feed additives, and less commonly utilized agro-industrial by-products. These limitations are not exclusive to the present investigation. The carbon footprints of individual feed ingredients may differ considerably depending on the emission-accounting methodology, system boundary definition, allocation approach, geographical origin, and underlying life cycle inventory datasets, while harmonized carbon footprint databases containing locally representative information on feed ingredients in China remain insufficiently developed [35]. More generally, variations in system-boundary definitions, dairy production systems, and GHG emission-accounting methodologies may also influence estimates of dairy farm emissions [36,37,38], and the establishment of more geographically representative GHG emission databases has been recognized as an important priority for the Chinese dairy industry [39].

These methodological limitations are especially pertinent to compound feeds, specialized feed additives, agro-industrial by-products, and imported feed ingredients, for which life cycle inventory data specific to individual suppliers and geographical origins are frequently unavailable. For internationally sourced feed ingredients, variations in agricultural production systems across countries, coupled with the limited availability of product-specific carbon footprint data, further restrict the accurate estimation of upstream GHG emissions based solely on farm-level purchasing records. Consequently, although incorporating upstream feed production emissions enables a detailed assessment of the dairy production system, the available information was insufficient to establish fully supplier-specific carbon footprints for all individual feed ingredients. In the sensitivity scenarios employing lower- and upper-bound emission factors, the estimated GHG emission intensity within the expanded system boundary ranged from 1.193 to 1.478 kg CO_2_-eq kg^−1^ FPCM, indicating that the absolute emission estimates were sensitive to the emission factors assumed for feed ingredients. Therefore, the expanded-boundary findings should be regarded as a sensitivity-based estimate reflecting system boundary expansion rather than a definitive supplier-specific product carbon footprint. Future studies would benefit from standardized methodologies for quantifying feed-related carbon footprints and improved accessibility of geographically representative, supplier-specific, and product-specific life cycle inventory datasets, particularly for feed ingredients obtained through international supply chains.

Within the predefined on-farm operational system boundary, lactating cows continued to represent the predominant source of enteric methane emissions, accounting for ap-proximately 60% of the total GHG emissions attributable to enteric fermentation. This finding highlights the critical role of nutritional management strategies, such as optimizing rumen fermentation efficiency, adjusting the forage-to-concentrate ratio, and employing methane inhibitors in emission reduction efforts [40,41,42].

In the present study, the analysis of farm dietary structure revealed that while the contents of dietary starch, CP, and RDP varied between years, none were correlated with enteric methane emissions. This result has two possible explanations: (1) Our analysis used annual averages of farm-level dietary composition, which may not accurately reflect the dynamic nutrient intake differences across various lactation stages and physiological states of the cows; enteric methane emissions are more closely related to actual nutrient intake and feeding conditions. (2) The regulation of methane emissions by dietary nutrition may be buffered by intermediate factors such as rumen microbial community structure and feed digestibility [43,44]. Using annual averages of single nutritional indicators may not capture these regulatory effects, leading to our finding of no statistically significant differences.

Furthermore, the observed decline in the proportion of lactating cows within the herd structure suggests an increase in the relative maintenance costs and emission contributions of the non-productive herd [45], which elevated the on-farm GHG intensity per unit of milk. The increase in on-farm energy consumption, including purchased electricity and directly recorded diesel use, reflected a potential mitigation gap in energy management during farm expansion and productivity enhancement. Farm scale was positively associated with energy consumption, likely because larger herds required greater use of ventilation, cooling, lighting, feed preparation, manure handling, and other mechanized operations. As noted above, the direct diesel category refers only to fuel consumed within dairy-farm operations and does not include fuel associated with upstream feed production.

Manure management was primarily driven by anaerobic digestion throughout the study period, demonstrating its environmental and economic value [8,46]. With increasing attention to environmental protection from both Chinese government agencies and the livestock industry [47], a more stringent manure management system has been gradually established in China. Some of the treatment methods adopted in the Chinese system differ from the typical manure management processes outlined in the IPCC guidelines. For instance, manure often undergoes solid-liquid separation initially. The solid portion may then be used for aerobic composting or dried for use as bedding in cattle housing, whereas the liquid portion is directed to storage ponds or subjected to anaerobic digestion. Such a split-stream approach alters the generation potential and emission pathways of methane and nitrous oxide while also enhancing the recovery and utilization of nutrients such as nitrogen and phosphorus and reducing their environmental release [48,49,50].

Nevertheless, this study did not calculate site-specific emission factors owing to China’s unique manure management modes, which represents a key limitation of the current work. Future studies should prioritize the development of region-specific emission factors tailored to Chinese dairy production systems by accounting for key environmental and management parameters, including temperature, moisture content, carbon-to-nitrogen ratio, aeration or turning frequency, storage duration, and surface-cover conditions. In the present study, the interannual variations in manure methane and nitrous oxide emissions were not driven by changes in manure treatment methods. Instead, they may have been associated with feeding management and DMI, a hypothesis supported by the correlation analysis, which identified links between manure GHG emissions and dietary starch content and DMI.

Within the on-farm operational boundary, the primary sources of excess emissions in HE farms are concentrated in three core areas: enteric methane from lactating cows, electricity consumption, and manure-related emissions. In contrast, LE farms had lower emissions by establishing more efficient production systems. First, they maintained a higher proportion of lactating cows, thereby reducing the emission redundancy associated with many non-productive animals [51,52] and enhancing the carbon emission efficiency of the herd structure. Second, they optimized feed utilization, achieving the target milk yield with less feed input, which lowered the emission intensity per unit of product [53,54]. These factors explain the results found in our correlation analysis, underscoring the value of multi-dimensional, synergistic optimization within the production system. Notably, LE farms surpassed HE farms in milk production during the later phase of the study while maintaining lower emission levels. This result demonstrates the feasibility of achieving high productivity and low GHG emissions. The underlying mechanisms possibly involve improved feed efficiency and optimization of the lactating herd structure. This provides a replicable practical model for large-scale dairy farms aiming to achieve a win-win scenario for both production and emission reduction. Furthermore, manure management practices were an important factor contributing to the differences in manure methane and nitrous oxide emissions between HE and LE farms. The proportion of manure treated by anaerobic digestion was associated with trends in manure-related GHG emissions. In 2021 and 2023, LE farms used anaerobic digestion to treat a higher proportion of their manure. Given that anaerobic digestion is a lower-emission technology [55], this may have contributed to lower methane and nitrous oxide emissions from manure [56]. The results of the present study identified mechanisms linking various production indicators to GHG emissions. The positive correlations between total herd size, energy consumption, and GHG emissions revealed that farm expansion and insufficient energy use efficiency are core drivers of emission increases. This finding suggests that mitigation efforts should address “controlling scale” and “improving efficiency.” This entails strategic planning of farm stocking density while enhancing energy use efficiency through measures such as promoting energy-saving equipment and optimizing operational workflows to reduce the energy-related GHG footprint per unit of output. The positive correlation between DMI and enteric methane emissions suggests that regulating intake patterns through methods such as optimizing diet palatability and implementing stage-specific precision feeding can reduce methane emission intensity from enteric fermentation. Several studies have documented this positive relationship, with methane yield typically ranging between 18.2 and 24.8 g methane per kg DMI [57,58,59]. Methane emissions are also influenced by dietary composition and nutrient content [59,60].

Furthermore, the negative correlation between the proportion of dietary starch and manure nitrous oxide emissions highlights a potential nutritional management pathway for manure emission reduction. This involves optimizing the dietary ratio of starch to crude protein to reduce the potential for fecal nitrogen conversion to nitrous oxide. Increasing the proportion of dietary starch can optimize rumen fermentation patterns, enhance microbial capture and the utilization of ammonia nitrogen (NH_3_-N), and improve microbial protein synthesis efficiency [61,62,63]. This shift allows more dietary crude protein to be converted into animal-usable protein forms, thereby reducing nitrogen excretion in feces and urine [33,64]. The positive correlation between feed efficiency and energy-related GHG emissions is possibly explained by the interaction between the production models associated with higher feed efficiency and their energy consumption patterns. Farms with higher feed efficiency typically use precise feed processing and delivery systems, alongside equipment for feed mixing, environmental control, and manure handling. The continuous operation of such equipment requires energy use in the form of electricity and diesel.

A principal strength of the present studies lies in the consistent application of a standardized on-farm operational system boundary to assess interannual variability and management-related differences in GHG emissions among 22 intensive dairy farms over three consecutive years. This methodological framework facilitates the identification of major GHG emission sources that can be directly addressed through targeted farm-level management interventions. Meanwhile, the supplementary sensitivity analysis incorporating upstream feed production emissions reveals that the overall GHG burden attributable to milk production increases substantially when emissions arising from upstream feed production processes are included. Accordingly, farm-level operational inventories and expanded-boundary assessments offer complementary analytical perspectives: the former is particularly valuable for identifying practical mitigation opportunities within dairy farm operations, whereas the latter provides a detailed estimate of the GHG emission burden associated with milk production per kg FPCM.

Future studies should incorporate supplier-specific primary life cycle inventory data covering feed production, processing, and transportation into farm-level operational GHG inventories to establish cradle-to-farm-gate assessments tailored to Chinese dairy production systems. Such integrated assessment frameworks would facilitate more accurate quantification of potential GHG mitigation opportunities throughout the dairy supply chain while preserving the practical relevance of operational emission inventories for identifying and implementing farm-level management strategies.

## 5. Conclusions

This study identified temporal patterns and management-related driving GHG emissions in Chinese dairy farms. Within the predefined on-farm operational boundary, enteric methane is the primary driver of GHG emissions in large-scale farms. Herd structure, production scale, energy consumption, and feed management are key determinants influencing farm-level emissions. Moreover, LE farms achieve synergistic development of high productivity and low emissions by optimizing the proportion of lactating cows, improving feed utilization, and adopting efficient manure treatment methods. In summary, green and low-carbon development of the dairy industry in North China can be promoted in large-scale operations through integrated strategies. These include precise adjustment of herd structure, regulation of nutrition and feeding practices, and optimization of manure management models.

The principal conclusions concerning interannual variability and the farm management characteristics distinguishing high-emission (HE) and low-emission (LE) dairy farms were derived from analyses consistently employing the predefined on-farm operational system boundary. The supplementary sensitivity assessment incorporating upstream feed production emissions demonstrated that expanding the GHG-accounting boundary increased the estimated emission intensity from 0.860 to 1.287 kg CO_2_-eq kg^−1^ FPCM, representing a 49.7% increase, with upstream emissions attributable to feed production contributing 33.2% of the total GHG burden under the expanded boundary. Accordingly, farm-level operational inventories and expanded-boundary assessments should be regarded as complementary rather than interchangeable analytical approaches. The former facilitates the identification of emission sources and management-related factors that can be directly addressed through on-farm interventions, whereas the latter offers a detailed estimate of the overall GHG burden associated with milk production.

Several priorities should be addressed in future studies. First, longitudinal studies with extended monitoring periods are needed to characterize temporal changes in GHG emissions and evaluate the long-term effects of management interventions and technological adoption on farm-level emission profiles. Second, the supplementary expanded-boundary assessment of upstream feed production emissions should be further improved by incorporating supplier-specific primary data concerning feed-crop geographical origin, fertilizer application practices, agricultural machinery use, feed-processing technologies, and transportation distances. Extending the system boundary to encompass downstream supply chain stages, including milk transportation, dairy processing, product distribution, and retail activities, would facilitate a cradle-to-consumer assessment of the environmental impacts associated with dairy production. Conducting such integrated assessments would necessitate stronger coordination between farm-level GHG inventories and operational data obtained from feed suppliers, dairy processors, and product distributors, supported where necessary by geographically representative feed emission factor databases and regional life cycle inventory datasets. Third, more studies of the principal emission hotspots identified in this study, including the interactions between dietary composition and enteric CH_4_ emissions and the effects of alternative manure management technologies on N_2_O emissions under different climatic conditions, would provide the mechanistic understanding required to develop targeted, region-specific GHG mitigation strategies.

## Figures and Tables

**Table 2:** Parameters for greenhouse gas emission estimation of manure management systems.

Item	MCF	EF_3_	Frac
Oxidation pond	0.76	0	0.35
3-month under-barn storage	0.28	0.002	0.28
Off-site field application	0.15	0.002	0.28
Anaerobic digestion	0.0438	0.0006	0.25

Abbreviations: MCF: methane conversion factor for manure management system; EF_3_: N_2_O emission factor within manure management system; Frac: fraction of manure nitrogen volatilized as ammonia (NH_3_). MCF, EF_3_ and Frac are dimensionless coefficients. For the oxidation pond, direct N_2_O emission is assumed to be negligible [20]. Manure N excretion was obtained by subtracting N retained in milk production and live-weight gain from feed N intake. A conversion factor of 6.38 was applied to milk protein concentration to derive milk N content [22]. Regression equations linking body protein content to cow body weight were used to estimate the N content attributable to live-weight gain [23,24,25].

**Table 3:** Emission factors of electricity consumption in different locations.

Location	EF (kgCO_2_/kWh)
Beijing	0.558
Tianjin	0.7041
Hebei	0.7252
Inner Mongolia	0.6849
Jilin	0.4932
Shandong	0.641
Henan	0.6058
Heilongjiang	0.5368

**Table 4:** Annual changes in farm production structure and performance indicators for 2021–2023.

Item	2021	2022	2023	SEM	*p*-Value
Herd structure:					
Total cows, head	3803	3993	4287	505.00	0.055
Lactating cows, head	1692 ^c^	1758 ^b^	1867 ^a^	218.92	0.013
Dry cows, head	237	270	260	37.37	0.257
* Replacement heifer, head	1874	1964	2161	252.26	0.186
Female calves (0–6 months)	475 ^b^	528 ^a^	593 ^a^	119.12	0.003
Lactating cow ratio, %	46.01 ^a^	44.68 ^b^	44.32 ^b^	0.476	0.002
Culled cows, head	668 ^b^	675 ^b^	886 ^a^	179.26	<0.001
Male calf sales, head	588	638	737	116.57	0.660
Milk production:					
Raw milk, kg/d	35.32	35.70	36.53	0.332	0.055
Milk fat, %	3.80 ^b^	3.78 ^c^	3.85 ^a^	0.021	0.001
Milk protein, %	3.27 ^b^	3.31 ^a^	3.28 ^b^	0.008	0.008
FPCM production, kg/d	34.35 ^b^	34.74 ^b^	35.78 ^a^	0.275	0.014
Diet composition, %:					
Forage	39.57 ^b^	40.28 ^ab^	41.04 ^a^	0.268	0.025
NDF	29.23	28.44	28.88	0.139	0.088
peNDF	19.06	19.10	19.06	0.145	0.975
Starch	26.69 ^c^	27.67 ^b^	28.23 ^a^	0.119	0.001
CP	16.74 ^a^	16.59 ^a^	16.35 ^b^	0.059	0.003
RDP	10.73 ^a^	10.36 ^b^	10.54 ^ab^	0.064	0.041
Feed consumption, t	41,408	47,428	44,535	5677	0.163
Feed consumption/head, t	11.46	11.66	10.57	0.201	0.130
DMI, kg/d	24.63 ^b^	24.72 ^b^	25.38 ^a^	0.139	0.011
Feed efficiency	1.45 ^b^	1.54 ^a^	1.58 ^a^	0.011	0.001
Energy consumption:					
Diesel, t	138.55 ^b^	191.83 ^a^	213.07 ^a^	22.859	0.001
Electricity, MWh	3294 ^b^	4481 ^a^	4657 ^a^	601	0.003

Abbreviations: DMI, dry matter intake; FPCM, fat- and protein-corrected milk; peNDF: physically effective neutral detergent fiber; RDP: rumen degradable protein. Forage is the concentration of ingredients in the diet, namely, 100% minus concentrates. a, b, and c indicate that the means are significantly different from each other at *p* < 0.05. * Replacement heifers encompass female calves aged 0–6 months; female calves are presented separately as a distinct physiological subgroup and must not be counted twice when determining the total herd population.

**Table 5:** Percentage of manure treatment methods (%) on dairy farms for 2021–2023.

Item	2021	2022	2023
Oxidation pond	3.34	3.34	3.34
3-month under-barn storage	4.55	4.55	4.55
Off-site field application	21.58	21.58	21.58
Anaerobic digestion	69.85	69.85	69.85

**Table 6:** Analysis of production structure and performance indicators for high- and low-GHG-emission farms for 2021–2023.

Item	2021		2022		2023	
	LE	HE	LE	HE	LE	HE
Herd structure:						
Total cows, head	5540	3611	2776	3570	3207	5171
Lactating cows, head	2569	1657	1282	1560	1424	2214
Dry cows, head	351	238	172	210	152	340
* Replacement heifer, head	2619	1717	1322	1800	1631	2617
Female calves (0–6 months)	746	459	373	501	500	700
Lactating cow ratio, %	46.55	45.50	45.79	44.28	45.22	43.28
Culled cows, head	1059	656	477	584	741	1043
Male calf sales, head	767	555	614	502	613	674
Milk production:						
Raw milk, kg/d	34.68	35.42	35.79	34.81	36.52	35.55
Milk fat, %	3.79	3.89	3.78	3.78	3.79	3.88
Milk protein, %	3.30	3.27	3.32	3.31	3.27	3.32
FPCM production, kg/d	33.70	34.82	34.86	33.86	35.46	35.01
Diet composition:						
Forage	39.56	39.45	40.90	40.35	40.26	40.70
NDF	28.80	29.80	28.41	28.70	28.75	28.67
peNDF	18.84	19.35	19.40	18.83	18.80	18.70
Starch	27.08	26.80	27.65	27.95	28.48	28.17
CP	16.85	16.73	16.38	16.75	16.52	16.28
RDP	10.66	10.78	10.37	10.10	10.56	10.48
DMI, kg/d	24.63	24.25	24.65	24.69	25.50	24.66
Feed consumption, t	56,997	42,486	28,511	44,338	32,590	53,447
Feed consumption/head, t	10.63	12.28	10.44	11.81	10.29	10.55
Feed efficiency	1.41	1.46	1.45	1.41	1.43	1.44
Energy consumption:						
Diesel, t	217.33	125.34	169.74	146.77	167.95	246.42
Electricity, MWh	5167	2980	3051	3779	2758	5823

Abbreviations: DMI, dry matter intake; FPCM, fat- and protein-corrected milk; peNDF, physically effective neutral detergent fiber; RDP, rumen degradable protein. Forage means the concentration of forage ingredient in the diet, namely, 100% minus concentrates. HE: high-greenhouse-gas-emissions group; LE: low-greenhouse-gas-emissions group. * Replacement heifers encompass female calves aged 0–6 months; female calves are presented separately as a distinct physiological subgroup and must not be counted twice when determining the total herd population.

**Table 7:** Percentage of manure treatment methods (%) for high- and low-GHG-emission farms during 2021–2023.

Item	2021		2022		2023	
	LE	HE	LE	HE	LE	HE
Oxidation pond	3.33	3.08	3.33	4.75	4.17	3.92
3-month under-barn storage	0	16.67	0	0	0	0
Off-site field application	1.67	27.47	17.50	0.25	0.83	11.64
Anaerobic digestion	95.00	52.78	76.67	95.00	92.50	84.44

HE: high-greenhouse-gas-emissions group; LE: low-greenhouse-gas-emissions group.

**Table 8:** Influence of system boundary expansion on GHG emission intensity.

Boundary	Included Emission Sources	GHG Intensity (kg CO_2_-eq kg^−1^ FPCM)	Contribution
On-farm operational boundary	Enteric CH_4_, manure CH_4_/N_2_O, direct electricity and fuel	0.86	66.8%
Feed upstream	Feed cultivation, processing, and upstream feed supply	0.427	33.2%
Expanded boundary	On-farm + feed upstream emissions	1.287	100%

## Data Availability

All datasets generated or analyzed throughout the present study are provided in the published article and its accompanying Supplementary Materials. The original raw datasets underlying the findings reported in this study have additionally been made available as supplementary files. Requests for further information concerning the detailed datasets and associated research materials may be addressed to the corresponding author.

## Supplementary Materials

The following supporting information can be downloaded at https://www.mdpi.com/article/10.3390/ani16203248/s1.
